# Assessment of scaling and corrosion potential of drinking water resources of Iranshahr

**DOI:** 10.1016/j.mex.2019.02.002

**Published:** 2019-02-05

**Authors:** Mahmoud Taghavi, Mohammad Hasan Mohammadi, Majid Radfard, Yadolah Fakhri, Safoura Javan

**Affiliations:** a Department of Environmental Health Engineering, School of Public Health, Social Development & Health Promotion Research Center, Gonabad University of Medical Sciences, Gonabad, Iran; b Department of Pediatrics, Zabol University of Medical Sciences, Zabol, Iran; c Department of Environmental Health Engineering, School of Health, Shiraz University of Medical Sciences, Shiraz, Iran; d Department of Environmental Health Engineering, School of Public Health and Safety, Shahid Beheshti University of Medical Sciences, Tehran, Iran; e Department of ٍEnvironmental Health Engineering, Neyshabur University of Medical Science, Neyshabur, Iran

**Keywords:** Corrosion potential, Water stability index, Langelier saturation index, Ryznar stability index, Puckorius scaling index, Aggressive index

## Abstract

The aim of this study was to evaluate corrosion and scaling potential of drinking water resources Iranshahr in order to considering necessary solutions to solve possible problems including internal corrosion of pipes, deterioration of water quality and reduce of water transfer capacity of distribution network system. The data showed that Langelier index ranged between −1.53 to −0.96, Ryznar index between 9.63–10.54, Aggressive index between 12.04 and 12.91, and Puckorius index between 9.05–10.68 for drinking water resources Iranshahr. Studied indices indicated that the drinking water in Iranshahr can be considered as corrosive.

## Graphical abstract

**Figure:**
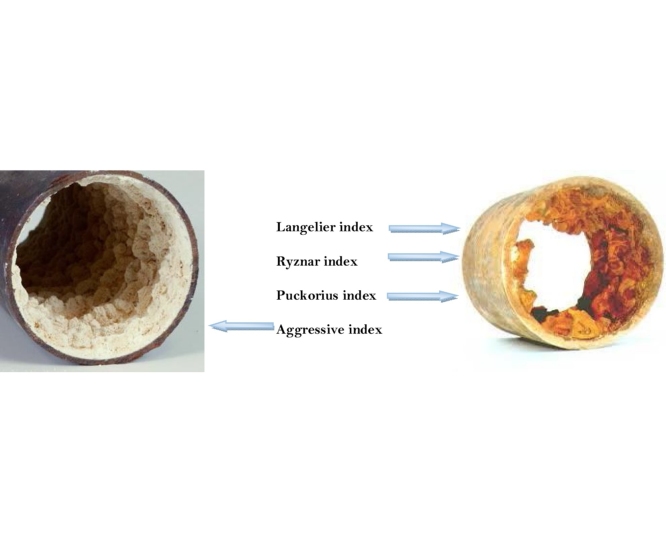

**Specifications Table**

**Table:** 

Subject area	Environmental Science
• More specific subject area:	• Drinking water chemistry
• Protocol name:	• Langelier index, Ryznar index, Aggressive index and Puckorius index
• Reagents/tools:	• DR5000 spectrophotometer,
• Experimental design:	• All physico-chemical parameters were determined according to standard methods mentioned in Standard Methods for the Examination of Water and Wastewater.
• Trial registration:	• No applicable
• Ethics:	• No applicable
• *Value of the Protocol:	• The data presented in this article can be useful for managers of water and wastewater companies, water resources facilities and treatment plants operators and operation manager of water distribution system. • Due to adverse health effect of corrosion potential of water resources as a results of solving of the materials and ingredient of pipes, ﬁttings and valves in distribution networks and its consequence on human health effect, assessment of scaling and corrosion potential of water in distribution network is necessary. • In dry climates, groundwater is almost the main source of drinking water, so, the continuous monitoring of the physic-chemical parameter of these valuable resources is very necessary. • Sources of drinking water in Iranshahr have corrosion potential and can threaten the health of consumers in the long term.

## Description of protocol

### Experimental design, materials and methods

#### Study area description

Iranshahr city in Sistan and Baluchistan province and its aquifers are located in South-East Iran between the latitudes 25˚15′N and longitudes 60˚45′E, encompassing an area of about 30,200 km^2^ (Fig. 6). The study area is a semi-flat plain region with a gentle slope toward the south has a warm, temperate climate. Also, the air’s highest and lowest temperatures are 50 °C and −6.2 °C, respectively, with an annual average of 26.8 °C.

#### Sample collection and analytical procedures

To calculate the corrosion indices, 36 samples of 1.5 l volume were collected from 18 sources, stored and transferred to lab according to standard method (stored in a dark cold box (4 °C) and transferred to laboratory within less than 3 h). The pH measured by pH meter device and water temperature determined by thermometer at the sampling points. Water quality parameters such as electrical conductivity, total dissolved solids, pH, calcium hardness, and alkalinity were measured in water samples in laboratory upon arriving samples. Alkalinity and hardness were determined using titration method and total dissolved solids was determined by gravimetric method. Sulfate was measured based on turbidity measurement at 420 nm by DR5000 spectrophotometer [1]. The equation of corrosion indices and their interpretations were summarized in Table 1. Calculation of indices was done using Microsoft Excel 2010.

**Table 1:** Summary of water stability indices in present study [2].

Index	Equation	Index value	Water condition
Langelier saturation index (LSI)	LSI = pH – pHs	LSI > 0	Water has super saturated condition and tend to precipitate CaCO_3_ in the system
	pHs = A + B – log (Ca^2+^) – log	LSI = 0	Water has saturated condition. It has no tendency to dissolve or precipitation of CaCO_3_
	(Alk) pH < = 9.3		
	pHs = (9.3 + A + B) – (C + D)	LSI < 0	Water is under saturated condition and tend to dissolve precipitated CaCO_3_
	(3) pH > 9.3		

Ryznar stability index (RSI)	RSI = 2pHs – pH	RSI < 6	Water has super saturated condition and tend to precipitate CaCO_3_ in the system
		6 < RSI < 7	Water has saturated condition. It has no tendency to dissolve or precipitation of CaCO_3_.
		RSI > 7	Water is under saturated condition and tend to dissolve precipitated CaCO_3_

Puckorius scaling index (PSI)	PSI = 2 (pHeq) – pHs	PSI < 6	Scaling likely will not occur
	pH = 1.465 + log	PSI > 7	It likely tend to dissolve scale
	(T.ALK) + 4.54		
	pHeq = 1.465 × log(T.ALK) + 4.54		

Aggressive index (AI)	AI = pH + log[(Alk)(H)]	AI > 12	It is non aggressive
		10 < AI < 12	It is moderately aggressive
		AI < 10	It is very aggressive

## Results

**Fig. 1:**
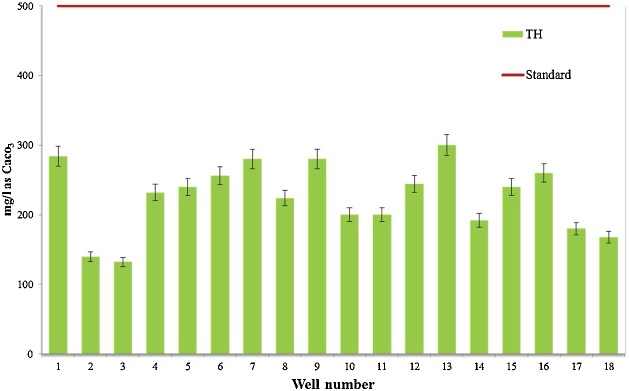
Compare total hardness with standard limit of Iran.

**Fig. 2:**
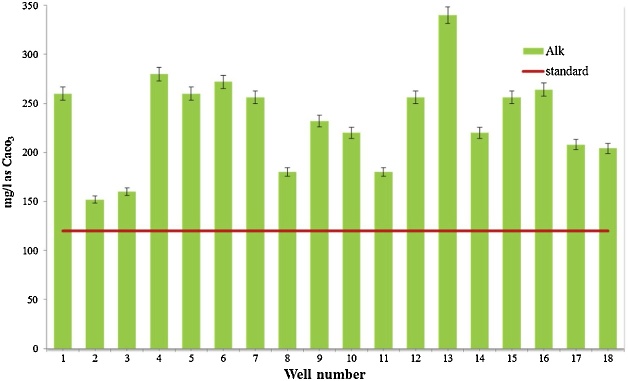
Compare Alkalinity with standard limit of Iran.

**Fig. 3:**
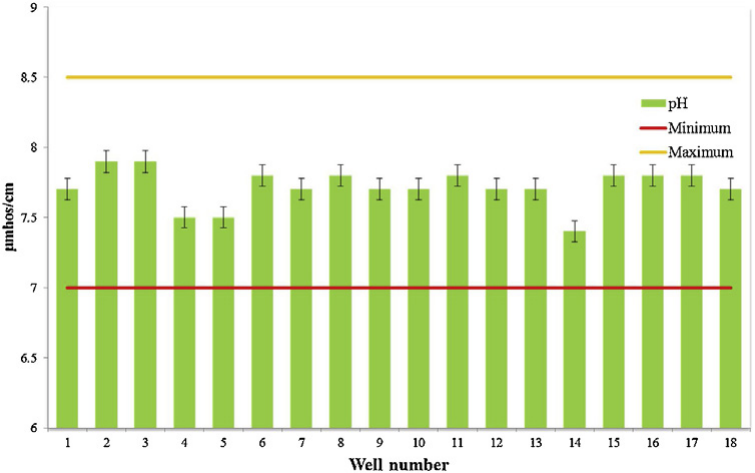
Comparison of pH with minimum and maximum standard limit of Iran (Desired limit).

**Fig. 4:**
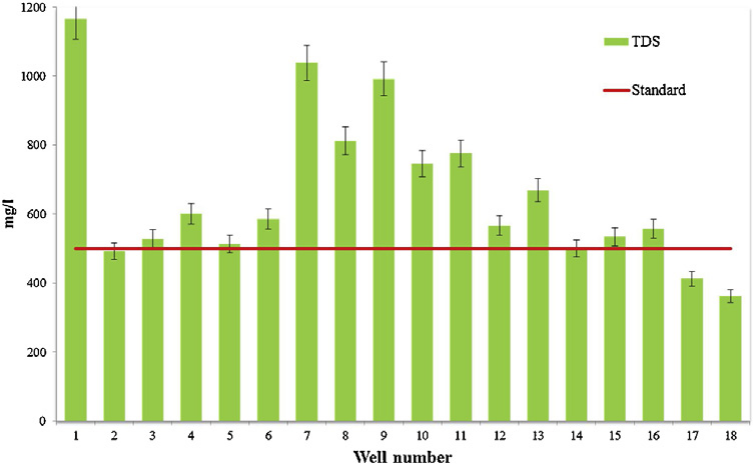
Compare TDS with standard limit of Iran.

**Fig. 5:**
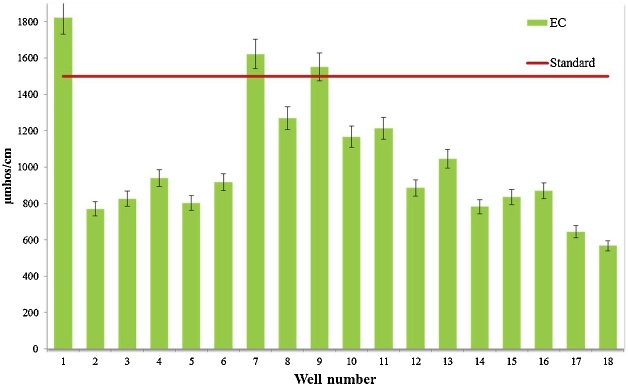
Compare electrical conductivity with standard limit of Iran.

Water quality assessment and safe drinking water supply is one of the most concerning issue in the world [3]. Data presented here deal with monitoring of physical and chemical parameter of drinking water in Iranshahr including pH, EC, TDS, Alkalinity, and EC which have shown in Table 2. The results of the calculations for Langelier, Ryzener, Puckorius and aggressive indices for drinking water resources of Iranshahr were presented in Table 3. Data on results of phsico-chemical parameter are compared with the National Iranian Water Standard (Fig. 1, Fig. 2, Fig. 3, Fig. 4, Fig. 5). Total hardness was equal to 225.11 mg/L as CaCO_3_ and was lower than the standard limit of Iran. The mean of electrical conductivity also was lower than it national standard. However, the mean of alkalinity and total dissolved solids were exceeded from standards. The mean of pH value was in the range of its desired values based on national standards of Iran. The data showed that Langelier index ranged between −1.53 to −0.96, Ryznar index between 9.63–10.54, Aggressive index between 12.04 and 12.91, and Puckorius index between 9.05–10.68 for drinking water resources Iranshahr. Based on all indices, drinking water in Iranshahr is corrosive.

**Table 2:** Mean and standard deviations values of physico-chemical parameters of distribution networks of Iranshahr city.

Parameter	Unit	Mean	SD	Max	Min	Standard
TH	mg/l as Caco_3_	225.11	49.52	300	132	500
ALK	mg/l as Caco_3_	233.33	47.59	340	152	120
T	°C	33.45	2.05	38.4	30.8	
pH	–	7.71	0.12	7.9	7.4	7-8.5
TDS	mg/l	658.33	222.2	1165	362	500
EC	(μmhos/cm)	1029.5	347.07	1821	567	1500

**Table 3:** Results of Water Stability indices of samples obtained from drinking water resources of Iranshahr city.

Well number	Index			
	LSI	RSI	AI	PSI
1	−1.13	10.3	12.52	9.92
2	−1.24	10.38	12.23	10.55
3	−1.32	10.54	12.22	10.68
4	−1.27	10.08	12.35	9.5
5	−1.25	10.01	12.3	9.45
6	−0.96	9.72	12.64	9.43
7	−1.27	10.22	12.53	9.83
8	−1.28	10.37	12.42	10.34
9	−1.31	10.28	12.47	9.95
10	−1.28	10.3	12.38	9.98
11	−1.38	10.51	12.31	10.41
12	−1.09	9.88	12.49	9.51
13	−0.98	9.63	12.68	9.05
14	−1.53	10.48	12.04	9.93
15	−1.02	9.8	12.46	9.49
16	−1	9.75	12.59	9.42
17	−1.1	9.98	12.35	9.82
18	−1.21	10.08	12.91	9.82
Mean	−1.20	10.13	12.44	9.84
Max	−0.96	10.54	12.91	10.68
Min	−1.53	9.63	12.04	9.05
SD	0.15	0.29	0.20	0.44

**Fig. 6:**
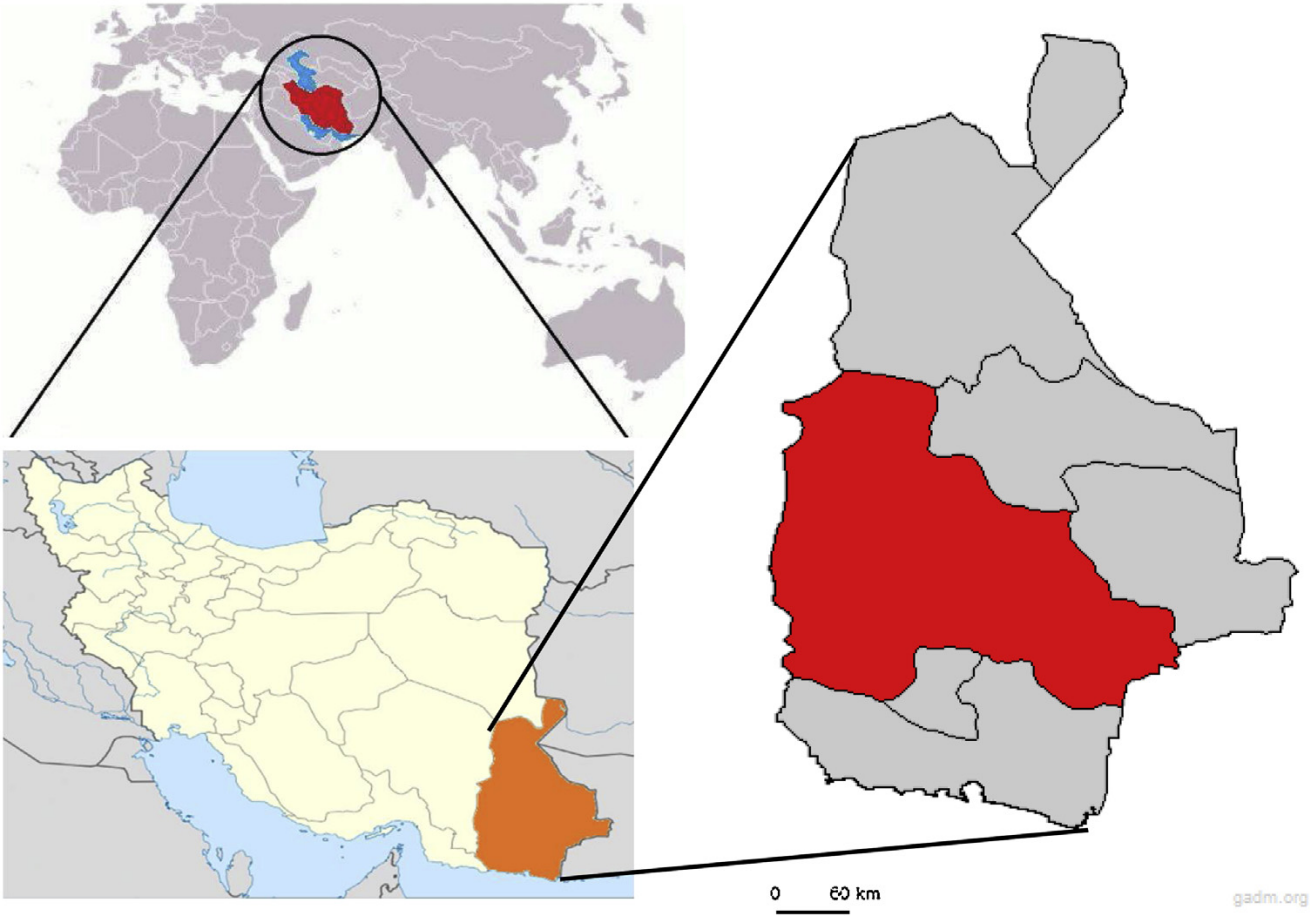
The study area and sampling location in Iranshahr city, Sistan and Baluchistan province, Iran.

## Concluding remarks

In the present study, it was attempt to evaluate corrosion and scaling potential of drinking water resources Iranshahr. Studied indices indicated that the drinking water in Iranshahr can be considered as corrosive. Total hardness and pH were well below the standard limit of Iran. However, electrical conductivity, total dissolve solids and alkalinity were exceeded form their standard values in some cases.

## Conflict of interest

The authors of this article declare that they have no conflict of interests.
